# The effect of foetal and early childhood growth on metabolic derangements of Sri Lankan children

**DOI:** 10.1186/s12887-022-03762-9

**Published:** 2023-01-10

**Authors:** V.P. Wickramasinghe, C. Arambepola

**Affiliations:** 1 Department of Paediatrics, Faculty of Medicine, University of Colombo, Kynsey Road, Colombo, Sri Lanka; 2 Department of Community Medicine, Faculty of Medicine, University of Colombo, Colombo, Sri Lanka

**Keywords:** Sri Lankan children, Body composition, Fat mass, BMI, Metabolic abnormality

## Abstract

**Background:**

Previous studies have shown that delayed prenatal growth followed by accelerated postnatal growth plays a significant role on the onset of adult metabolic diseases. The present study aimed to identify the effects of intrauterine and later growth on metabolic derangements among children in Colombo, Sri Lanka.

**Methods:**

A school-based cross-sectional study was conducted among 5–15-year-old children selected using a two-stage probability-proportionate-to-size cluster sampling technique. Birth-weight (BW) was extracted from records (denotes prenatal growth) and body mass index(BMI)-Z score and fat mass(FM) measured to denote the current growth of children. Fasting and random blood glucose, lipid profile and blood pressure(BP) were measured. The sample was stratified by age (5—< 10 and 10—15 years); was further categorized into tertiles of BW and BMI-Z scores. Based on these two parameters, metabolic parameters were evaluated within each age category.

**Results:**

The sample comprised 833(494 boys) healthy school children. Metabolic parameters did not significantly differ by sex or across BW tertiles of each BMI-Z score tertile. However, significant changes in some metabolic parameters were noted across the BMI-Z score tertiles of each BW tertile. Children belonging to the lowest BW and highest BMI-Z score tertiles had worst metabolic profiles, while those in the lowest BW as well as BMI-Z score tertile were protected. Excessive fat deposition seemed to move children to higher BMI-Z score tertiles.

**Conclusion:**

Poor prenatal growth is not the sole risk factor for abnormal metabolic profile found in childhood. Those who gain fat, denoted by weight, during early childhood are at a higher risk of developing metabolic abnormalities than those who do not. This favours the accelerated postnatal growth hypothesis.

**Supplementary Information:**

The online version contains supplementary material available at 10.1186/s12887-022-03762-9.

## Background

Physical growth distinguishes paediatric population from adults. Not only it reflects the nutritional status of an individual but also determines his/her future health. This has given insights to the origin of non-communicable diseases (NCD) in adults and introduces new avenues in prevention of these illnesses.

Many retrospective epidemiological studies have shown the impact of early nutrition on later health [1]. Barker noted that small-for-gestational-age (SGA) individuals were the most disadvantaged as far as the development of many NCDs are concerned [1]. Their foetal as well as early postnatal under-nutrition has been noted to have many adverse outcomes later in life with respect to blood pressure (BP), cardiovascular diseases (CVD), impaired glucose homeostasis, abnormal lipid metabolism, hepatic steatosis and even malignancy. This marked the beginning of the “fetal origin of adult diseases” hypothesis, in which under-nutrition during early life was seen as a threat for development of NCD later in life.

Although Barker’s hypothesis states that the risk for NCDs are higher among low BW babies data from India has shown that if they remain thin or underweight at 16 to 19 years of age, the risk for elevated BP is minimum compared to normally nourished counterparts [2].

Rapid growth in pre-term children has shown to lead to many metabolic derangements such as high BP, cholesterol, insulin and leptin resistance with significant endothelial dysfunction at 16 years of age compared to their counterparts having a slower growth [3, 4]. Similarly, when SGA infants at-term were given enriched formula to promote growth, they had shown elevated BP and increase in fat mass(FM) at 8 years of age [5]. This acceleration has also shown to lead to central adiposity in many, leading to develop CVD and type 2 diabetes mellitus (DM) later in life [6]. These adverse health outcomes observed with “over feeding” as opposed to under nutrition in the immediate postnatal period, led Singhal & Lucas to propose the “accelerated postnatal growth” hypothesis [3].

It is shown that there could be a period in fetal and/or early postnatal life where “programming” of growth takes place, which would begin to “track” to adult life [7, 8]. Once tracking has established, changing the growth trajectory by offering more protein and energy may lead to storing the excess as fat, thus resulting in obesity rather than letting an individual grow with an appropriate body composition. Asian populations, where low BW cohorts are found to be more vulnerable to NCDs, in the backdrop of drastic nutritional and socio-economic transitions that have transformed the traditional behaviours practices of Asian societies into those accumulating many NCD risk factors.

Growth is a continuous process and birth is only an event in that process that changes the environment in which they grow. In line, post-natal growth needs to match its fetal growth, so that adverse programming effects are not encountered. This suggests that managing growth faltering must be done cautiously. Such proper management of growth will yield an optimum programming effect in children, which will exert better long-term effects when applied at a critical period during early growth (prenatal or early postnatal period) [9]. This study was carried out to identify the effect of intrauterine growth (reflected by BW) and later growth (reflected by BMI-Z scores in early and late childhood) on metabolic derangements among 5–15-year-old children in Colombo, Sri Lanka.

## Methods

A cross-sectional study was carried out among 5–15-year-old apparently healthy Sri Lankan children recruited from 15 state schools in the district of Colombo, Sri Lanka. They represented children studying in grades 1–10 and of mixed ethnic and socio-economic backgrounds. Those with any acute/chronic illness or on any medication, as confirmed by documental evidence were excluded. The minimum sample calculated was 790 to ensure detection of an expected proportion of children with obesity of 2% [10]; with level of precision of 0·01; confidence interval of 0·05; and a non-response rate of 5%. The sample was recruited using a two-stage, probability-proportionate-to-size (PPS) cluster sampling technique. Stratified by age and sex, one class from each grade was included as a cluster, which was randomly selected from each school that was selected according to PPS. The eligible students and their parents were informed about the procedure, and written consent to participate from parents/legal guardian of minors and assent from children above 12 years were obtained. The Ethics Review Committees of the Faculty of Medicine, University of Colombo and Lady Ridgeway Hospital for Children approved the study. After obtaining permission from school principals, the parents of the students were invited to participate.

### Assessment of growth

During data collection, BW was extracted from the child health development record as a proxy measure of their foetal growth. Body mass index (BMI), waist circumference (WC) and body FM were considered as proxy measures of the current growth of children.

Height and weight were measured using a standard protocol [11] in order to calculate the BMI (weight (kg)/ height (m)^2^). Based on IOTF classification, BMI categorization was made. BMI-Z scores were calculated according to WHO standards (2007). Waist circumference was measured at the midpoint between the lower border of palpable rib and upper border of the iliac crest in the mid axillary line, using a non-stretchable flexible tape to the nearest 0.1 cm at the end of expiration.

Total body FM of children was assessed using the whole-body bio-impedance assay (BIA) technique using eight-electrode InBody 230® machine (Boispace Co Ltd, South Korea). This technique had been previously validated against a Sri Lanka-based BIA Eq. [12] and height-weight based Eq. [13]. Assessment compared with BIA Eq, correlations of 0·95 (*p* < 0·001) and 0·97 (*p* < 0·001) were shown with total body water (TBW) and fat free mass (FFM), respectively. Also, when compared with assessments made by height-weight based equation, high correlations were given for both TBW (*r* = 0·91; *p* < 0·001) and FFM (*r* = 0.93; *p* < 0·001). The FM and FFM were evaluated in absolute amount (kg), as an index (FMI, FFMI kg/m^2^) and as a percentage out of total body weight (%FM).

### Assessment of metabolic derangements

Bio-physical measurements were done as parameters to detect metabolic derangements. The BP was measured adopting standard practice defined by Task Force Report on High Blood Pressure in Children and Adolescents (1996), using a mercury sphygmomanometer in seated position with arm placed at the levels of the heart and resting on the arm of the chair to relax the muscles, after a 10-min rest [14]. The first and fifth Korotkoff sounds were used to represent the systolic BP (SBP) and diastolic BP (DBP), respectively. If elevated blood pressure was noted, it was rechecked after a 30-min rest [14]. Blood pressure was evaluated using Z scores of UK standards and elevated blood pressure defined as >  + 2 Z scores from mean for age and sex for both SBP and DBP [15].

Blood was drawn after a 12-h overnight fast to test for fasting blood glucose (FBS) and lipid profile. Oral glucose tolerance test was also done after giving a drink of anhydrous glucose 1·75 g per kg per body weight to a maximum of 75 g; and blood drawn two hours later for random blood sugar (RBS).

### Biochemical analysis

Biochemical analysis was performed at the endocrine laboratory of the Obstetrics and Gynaecology department, University of Colombo. Blood glucose was assessed by enzymatic spectrophotometric method using glucose oxidase and peroxidase enzymes. Quantitative analysis was done using spectrophotometer (BioSystems®). Cholesterol ester molecule was cleaved using cholesterol oxidase and peroxidase enzymes and cholesterol level was assessed quantitatively using spectrophotometer (BioSystems®). Enzymatic cleavage of triglycerides (TG) was done using glycerol phosphate oxidase and peroxidase enzymes and end-product was assessed quantitatively by spectrophotometer (BioSystems®). HDL-cholesterol (HDL-c) was measured using enzymatic spectrophotometry with enzymatic analysis using cholelseterol esterase, cholesterol oxidase and peroxidase. Quantitative assessments were done by spectrophotometer (Randox ®). LDL-cholesterol (LDL-c) was calculated using the equation (total cholesterol—(HDL-c + TG/5)). Serum insulin was assessed by **s**olid phase, enzyme-labelled chemiluminescent immunometric assay using immulite 1000 ®analyser (Siemens, USA).

### Statistical analysis

Quantitative data were described using mean and standard deviation (SD) and qualitative data using proportions. The sample was stratified by age into two groups: 5- < 10 years and 10–15 years in keeping in line with IDF classification for obesity related metabolic derangements [16]; and each group further categorized into tertiles of BW (denoting fetal growth) and BMI-Z scores (denoting current growth). Metabolic parameters within each age category were evaluated according to different BMI-Z score and BW tertile combinations. For this purpose, 3 × 3 tables were constructed, and their significance assessed using ANOVA (for normally distributed variables) and Kruskal–Wallis (for non-normally distributed variables) tests at 0.05 level of significance.

## Results

A total of 833 (494 boys) children were studied. Characteristics related to the growth of the children stratified by age and sex are presented in Table 1. In both 5- < 10- and 10–15-year age categories, the weight, BMI, FM, FMI and WC were significantly higher in girls, while FFMI was higher in boys. BW was similar in all four groups. Further, the overall prevalence of obesity was 3.2%; overweight was 9.6%; and severe wasting has been 8.9% in the study sample (Table 2). Descriptive statistics of the anthropometric and body composition parameters are given in Supplementary Tables 1a -c.

**Table 1:** Demographic and growth parameters of the study population according to age category and sex

Characteristic	5 – < 10-year age group			10 – 15-year age group	
	**Male (*N* = 239)**	**Female (*n* = 181)**	**Male (*n* = 255)**		**Female (*n* = 158)**
	Mean(SD)	Mean(SD)	Mean(SD)		Mean(SD)
Age (years)	7.8(1.3)	7.9(1.3)	12.3(1.5)		12.6(1.5)
Birth weight	3.0(0.5)	3.0(0.6)	3.0(0.5)		2.9(0.5)
Height (cm)	124.2(8.9)	125.0(9.9)	146.8(11.4)		148.2(9.8)
Weight (kg)	23.1(6.4)	24.9(8.5)*	36.5(10.8)		39.8(12.6)*
BMI (kg/m^2^)	14.8(2.5)	15.6(3.3)*	16.6(3.3)		17.8(4.3)*
Height Z-score	-0.32(1.0)	-0.25(1.1)	-0.71(1.1)		-0.6(1.2)
Weight Z-score	-0.8(1.4)	-0.32(1.6)*			‡
BMI Z-score	-1.02(1.5)	-0.7(1.8)	-1.02(1.7)		-0.6(1.7)*
Fat mass (kg)	4.4(3.7)	6.2(4.8)*	7.2(5.4)		11.4(8.0)*
Fat free mass (kg)	18.7(3.8)	18.7(4.5)	29.2(7.8)		28.4(6.3)
Fat mass index (kg/m^2^)	2.8(2.0)	3.7(2.5)*	3.3(2.3)		5.1(3.3)*
Fat free mass index (kg/m^2^)	12.0(1.2)	11.8(1.4)	13.3(1.8)		12.8(1.7)*
% FM	17.5(9.1)	22.2(9.8)*	18.7(8.9)		26.2(10.8)*
Waist Circumference (cm)	53.6(7.5)	56.1(8.8)*	61.9(9.7)		64.8(10.7)*

^*^ *p* < 0.05 when compared each parameter between gender groups within each age category

^‡^ SD Scores not calculated for that age group

**Table 2:** Categorization of the study population according to IOTF cutoff values according to age category and sex

Nutritional status	5 – < 10-year age group		10 – 15-year age group		Total no. (%)
	**Male No. (%)**	**Female No. (%)**	**Male No. (%)**	**Female No. (%)**	
No	239	181	255	158	833
Obese	8 (3.3%)	9 (4.9%)	6 (2.3%)	4 (2.5%)	27 (3.2)
Overweight	14 (5.8%)	21 (11.6%)	19 (7.4%)	26 (16.4%)	80 (9.6)
Normal	88 (36.8%)	73 (40.3%)	100 (39.2%)	65 (41.1%)	326 (39.1)
Thinness					
Thinness 1	71 (29.7%)	34 (18.8%)	70 (27.4%)	27 (17.1%)	202 (24.3)
Thinness 2	36 (15.0%)	26 (14.4%)	42 (16.5%)	20 (12.6%)	124 (14.9)
Thinness 3	22 (9.2%)	18 (9.9%)	18 (7.1%)	16 (10.1%)	74 (8.9)

Table 3 shows the distribution of metabolic parameters of the total sample by tertiles of the current BMI-Z score and BW. Since there was no sex difference noted in relation to each metabolic parameter(data not shown), both males and females were analysed together according to age groups. When compared along each BW tertile most of the parameters showed deterioration except for FBS and LDL-c in younger age group and FBS, total cholesterol and HDL-c in older age group. However, when the parameters were compared across different BW tertiles of each BMI-Z score tertile, no significant difference was noted.

**Table 3:** Distribution of the metabolic parameters according to birth weight (denoting fetal growth) and BMI-Z score (denoting current growth) tertiles and age category of children

Birth weight tertiles	5—< 10-year age group				10—15-year age group			
	**Current BMI Z score tertile**			**Total**	**Current BMI Z score tertile**			
	**T1**	**T2**	**T3**		**T1**	**T2**	**T3**	**Total**
	Mean(SD)				Mean(SD)			
	**a Systolic blood pressure Z score**							
**T1**	-1.95(1.1)	-1.92(1.3)	-1.38(1.5)	-1.80(1.2)	-1.47(1.1)	-1.07(1.0)	-0.51(1.1) ^1^	-1.10(1.1)
**T2**	-2.50(1.2)	-1.75(1.0)^3^	-1.41(1.4)^1^	-1.90(1.3)	-1.70(1.1)	-0.88(1.1) ^3^	-0.37(1.1)^1^	-0.96(1.2)
**T3**	-2.18(1.1)	-1.74(1.1)	-1.17(1.0)^1,2^	-1.60(1.2)	-1.50(1.1)	-0.98(0.99)	-0.65(1.3) ^1^	-0.97(1.2)
**Total**	-2.18(1.1)	-1.81(1.1)	-1.30(1.1)		-1.54(1.06)	-0.97(1.02)	-0.52(1.17)	
	**b Diastolic blood pressure Z score**							
**T1**	-0.22(0.88)	-0.11(0.99)	0.34(1.1)^1^	-0.04(1.0)	0.47(0.9)	0.78(0.8)	1.24(0.8) ^1^	0.75(0.92)
**T2**	-0.40(0.94)	-0.14(1.00)	0.30(1.2)^1^	-0.05(1.1)	0.44(0.99)	1.10(0.82)^3^	1.31(0.95) ^1^	0.96(0.98)
**T3**	-0.09(0.85)	-0.01(1.1)	0.44(1.1)	0.15(1.1)	0.55(1.1)	0.79(1.0)	1.19(0.93) ^1^	0.90(1.00)
**Total**	-0.25(0.90)	-0.04(1.03)	0.36(1.13)		0.48(0.98)	0.89(0.91)	1.24(0.91)	
	**c Fasting blood sugar**							
**T1**	80.5(7.8)	78.6(9.4)	89.5(9.5)	79.6(8.8)	82.3(7.9)	80.5(6.9)	80.7(9.7)	81.3(8.1)
**T2**	77.5(8.5)	78.6(10.7)	78.8(9.7)	78.3(9.6)	80.7(8.8)	83.5(7.9)	83.7(8.6)	82.7(8.5)
**T3**	77.3(8.8)	77.6(8.5)	78.9(6.6)	78.1(9.6)	83.9(6.5)	82.0(8.8)	84.8(7.9)	83.6(8.0)
**Total**	78.6(8.3)	78.3(9.5)	78.3(9.5)		82.2(7.9)	82.2(8.0)	83.3(8.7)	
	**d Random blood sugar**							
**T1**	87.6(13.6)	89.4(14.6)	93.3(18.5)	89.6(7.7)	86.7(13.8)	89.9(14.8)	92.6(21.0)	89.2(16.2)
**T2**	81.9(19.5)	82.4(15.0)	93.2(16.8)^1,2^	85.7(17.9)	85.5(19.6)	88.8(16.3)	90.8(16.3)	88.4(18.6)
**T3**	83.4(13.5)	84.4(15.0)	91.4(15.3)	87.2(15.1)	90.4(19.3)	90.9(19.2)	97.2(12.8)	93.5(17.0)
**Total**	84.5(16.0)	85.7(15.1)	92.4(16.5)		87.2(17.1)	89.9(18.0)	94.1(16.4)	
	**e Total cholesterol**							
**T1**	155.7(43.1)	160.4(31.8)	173.5(29.7)	161.7(36.6)	162.7(31.6)	155.9(38.0)	171.2(33.5)	162.8(34.3)
**T2**	167.3(31.0)	168.5(32.2)	175.3(36.8)	170.3(35.0)	166.5(33.4)	151.5(36.9)	166.2(37.3)	160.9(36.4)
**T3**	157.9(25.3)	162.0(38.3)	172.3(34.4)	165.4(34.1)	167.9(31.9)	167.2(33.1)	171.2(33.8)	169.1(32.9)
**Total**	161.6(35.3)	162.3(35.7)	173.6(33.9)		165.2(32.1)	158.2(36.3)	169.6(34.7)	
	**f LDL-c level**							
**T1**	88.9(43.5)	95.9(32.5)	108.0(30.8)	96.0(37.4)	95.2(32.8)	93.5(40.3)	109.5(34.7)	98.3(36.0)
**T2**	103.2(34.4)	104.2(40.6)	112.3(38.8)	106.7(37.9)	102.8(32.9)	88.0(35.4)	97.6(71.7)	95.8(34.0)
**T3**	107.2(25.6)	98.7(33.6)	113.5(35.1)	107.4(32.9)	97.8(32.0)	105.2(36.4)	107.1(33.4)	104.2(34.1)
**Total**	98.6(37.1)	99.7(35.6)	111.9(35.3)		98.2(32.6)	65.6(37.6)	104.6(33.9)	
	**g Triglyceride level**							
**T1**	65.4(26.8)	67.2(19.3)	88.8(39.1) ^1,2^	70.7(28.9)	74.9(30.2)	80.0(25.9)	94.4(40.7) ^1^	81.3(33.7)
**T2**	71.1(31.8)	73.3(24.8)	77.0(35.8)	73.7(31.0)	75.5(30.0)	79.1(29.0)	100.6(46.9) ^1,2^	84.9(37.6)
**T3**	63.3(18.5)	65.6(28.1)	75.6(28.9)	73.7(31.0)	75.3(36.9)	81.9(46.4)	90.4(37.4)	83.6(40.8)
**Total**	66.9(26.9)	68.6(24.1)	77.9(33.9)		75.2(31.8)	80.4(35.9)	94.7(41.5)	
	**h HDL-c level**							
**T1**	53.7(17.3)	50.9(19.3)	47.7(18.3)	51.3(16.6)	52.6(18.5)	46.4(15.1)	42.8(12.3)^1^	48.3(16.5)
**T2**	50.8(18.1)	49.2(15.2)	47.5(12.9)	49.1(16.4)	48.6(14.9)	46.4(13.7)	48.4(16.3)	47.7(14.9)
**T3**	46.1(15.3)	43.7(11.9)	43.1(11.9)	44.3(13.3)	53.7(22.6)	45.6(14.8)	45.9(13.9)	47.6(16.9)
**Total**	49.8(17.0)	48.9(15.1)	45.9(14.8)		51.6(18.6)	46.1(14.4)	45.9(14.4)	

^1^T3 significantly differ from T1; ^2^T3 significantly differ from T2; ^3^T2 significantly differ from T1

In both age groups, both mean SBP-Z score and DBP-Z score, when compared across different BMI-Z score tertiles, BP-Z scores increased within each BW tertile (Table 3a & 3b). Characteristically, the lowest SBP-Z score and DBP-Z score values of both age groups were found in the lowest current BMI-Z score tertile. Children who were in the lowest BW tertile had a lower SBP-Z score and DBP-Z score when they remined thin (lowest current BMI-Z score tertile) compared to their counter parts who were heavier (highest BMI-Z score tertile).

The mean FBS did not show any significant differences when compared across different BW or BMI-Z score tertiles (Table 3c). RBS also showed increasing values across BMI-Z score tertiles, but a significant increase was seen only in the middle BW tertile in the younger age group (Table 3d).

Both total cholesterol and LDL-c did not vary significantly either across the BW tertiles or BMI tertiles (Table 3e & 3f). However, TG values increased with the increase in the current BMI-Z score tertiles. Lowest TG values were noted in the lowest current BMI-Z score tertile and lower BW did not increase the risk of having higher TG levels unless they increase the weight later in life (Table 3g). Similarly, HDL-c levels decreased with increasing in the BMI-Z score tertiles in the younger age group. In the older age group this pattern was seen only among those of the lowest birth weight tertile. Higher HDL-c levels were seen in the lower current BMI-Z score tertiles and lower BW tertiles (Table 3h).

The percentage FM differed significantly across the BMI tertiles, with higher values demonstrated in the highest BMI-Z score tertile compared to the other two tertiles, demonstrating an almost two-fold increase across BMI-Z scores (Table 4). This suggests that the gain in weight (or BMI) was most likely to have occurred due to an accumulation of fat rather than a growth maintaining a healthy body composition.

**Table 4:** Distribution of the fat distribution according to birth weight (denoting fetal growth) and BMI-Z score (denoting current growth) tertiles and age of the children

Birth weight tertiles	5—< 10-year age group				10—15-year age group			
	**Current BMI Z score tertile**				**Current BMI Z score tertile**			
	**T1**	**T2**	**T3**	**Total**	**T1**	**T2**	**T3**	**Total**
	Mean(SD)				Mean(SD)			
**T1**	13.1(5.0)	15.1(4.3)	28.1(9.6)^a,b^	17.4(8.6)	15.1(6.2)	19.5(5.8)^3^	33.6(10.2)^a,b^	21.4(10.4)
**T2**	131(3.4)	16.5(4.2)	30.5(9.3)^a,b^	19.7(9.7)	16.4(7.4)	17.2(5.2)	32.0(9.6)^a,b^	21.7(10.3)
**T3**	15.1(6.7)	16.4(3.8)	28.9(10.5)^a,b^	21.5(10.3)	12.8(4.0)	19.1(7.4)^c^	29.8(9.1)^a,b^	22.0(10.3)
**Total**	13.6(5.1)	15.9(4.2)	29.2(9.8)		15.0(6.3)	18.5(6.3)	31.5(9.6)	

^a^T3 significantly differ from T1

^b^T3 significantly differ from T2

^c^T2 significantly differ from T1

## Discussion

Our study confirms that BW in isolation would minimally affect the metabolic abnormalities related to NCD in children. Consequently, children with optimum body composition (children belonging to middle BW and BMI-Z score tertiles) were found to be metabolically healthier, denoting the role of both BW and post-natal growth on subsequent metabolic derangements. However, more importantly and contrary to the Barker hypothesis, the study further shows that, if a child is born with a lower BW but tend to have a lower BMI-Z score during childhood, they are protected from adverse metabolic derangements. This contrasts with children born with a lower BW, followed by a higher weight gain during childhood (those belonging to lower BW and higher BMI-Z score tertiles) showing the worst metabolic outcomes. This highly suggests that BW is not the only risk factor that determines a poorer metabolic profile in children, but the weight gain during the first few years of life has a significant contribution to the development of an adverse metabolic profile.

Blood pressure appears to track from a younger age and the 1970 British birth cohort showed an inverse relationship between SBP at 10 years of age and BW [17]. This study showed that the highest mean SBP was observed among those belonging to the lowest BW and highest current weight tertile, while the lowest mean SBP was seen in the highest BW and lowest bodyweight tertile. In comparison, the best mean SBP-Z score and DBP-Z score observed in our study were among children belonging to the mid BW and lowest current BMI-Z score tertiles. This reflects that both poor growth during fetal period as well as excess growth during post-natal period could be risk factors for development of high BP later in life. Furthermore, Barker and co-workers showed an inverse relationship of SBP with mothers’ height, which could be considered as an indirect measure of uterine size that may contribute to birth size of her offspring [17]. Although we have not looked at this relationship, our data shows that optimum birth size provides protection from future metabolic derangements. Therefore, could postulate that the control of NCDs could even be a generation long process, where a healthy girl child with good uterine size would give birth to a well-grown healthy baby.

FBS and RBS 2 h after a glucose load failed to show a clinically relevant relationship, highlighting its poor applicability as a routine screening test to detect impaired glycaemic control. A previous study showed that fasting and 2-h post glucose serum insulin levels as well as insulin resistance measured using HOMA-IR had a strong relationship with BW and BMI [18]. Hales and co-workers studying a group of 59–70 year old men from Hertfordshire UK, showed that adults with a low BMI, helped in protecting those with poor prenatal growth against dysglycaemia [19].

It is shown that children with “catch-up growth” have a greater risk of dying from coronary heart diseases (CHD) later in life [20]. The Helsinki study showed that the highest death rates were seen in children who were thin at birth but caught up their weight to the level of average child or above at 7 years of age [20]. Similarly, Harvard growth study showed the effect of high BMI at childhood on CHD in later life, which was independent of adult BMI [21]. In comparison, our data also showed that low BW children achieving a higher BMI in early life would have an adverse metabolic profile. As to the cause underlying this, accumulation of fat was strongly implicated. Prader and co-workers originally defined ‘catch up growth” as a phenomenon that occurs after slowing of growth following illness or starvation, that recovers after correction of the insult [22], Loose application of this on low birth weight babies, would have had led to dire consequences. However more clear knowledge at present day had differed the use of this practice on feeding to achieve a “catch up” growth for low birth weight children as it would lead to harmful effects of “accelerated growth” [4].

Reports suggest that fetal nutrition, as denoted by BW, may have an inverse programing effect on abdominal adiposity in later life, which could contribute to the development of insulin resistance [6]. This indicates that one’s body composition during fetal and early life is associated with adult disease risk [23].

It is shown that rapid weight gain during infancy in SGA children is associated with increased FM rather than FFM [6, 24]. Early “catch-up growth” following SGA birth has been noted as a CVD risk factor in later life rather than SGA alone [25]. However, the tendency of SGA children to assimilate intra-abdominal fat is not yet clear; whether due to low BW itself, rapid postnatal “catch-up” growth or a combination of both [6, 26]. During recovery from wasting or protein-energy malnutrition in children and adults, FM is shown to accumulate much faster than the muscle mass. This phenomenon could partly explain the adverse outcomes in SGA children during “catch-up” growth [6]. Therefore, although “catch-up” growth explicitly confers several benefits in relation to improved neurodevelopment, enhanced immune function, and achieving adult height, there are certain adverse metabolic consequences as well, such as the insulin resistance, metabolic syndrome, DM, CVD, increased fat mass and obesity. As such, it is imperative that early feeding of SGA children requires close growth monitoring to achieve an optimum body composition.

This paper highlights the importance of improving the clinical practice related to children in early life, especially in developing countries where poor prenatal growth of a child is still a grave issue. In such clinical settings, measures should be in place to prevent excess weight gain during early childhood in SGA children. Length/height of a baby, which is usually parallel to weight gain, should be assessed regularly at 3–6-month intervals, so growth could be evaluated in a manner where weight is standardize to height (use of length/height for weight or BMI for age charts).

## Conclusion

Children who show a rapid growth during early childhood, especially SGA babies, are at a higher risk to develop NCD related abnormal metabolic profile in childhood. Those who gain fat, denoted by weight, during early childhood are at a higher risk of developing metabolic abnormalities than those who do not, denoting that poor prenatal growth is not the sole risk factor to develop NCD later in life. This favours the accelerated postnatal growth hypothesis. Therefore, maintaining optimum growth appropriate to the birth weight is important to prevent NCD later in life.

Larger cohort studies are needed from developing countries to understand the best possible trajectory of growth based on anthropometry with the metabolically favourable body composition to prevent complications of SGA infants.

## Supplementary Information

**Additional file 1.**

## Data Availability

Data will not be made available in a public repository as we have not obtained ethical clearance to share data publicly. However, on request from corresponding author data could be provided while maintaining anonymity.

## Abbreviations

ANOVA: Analysis of variance
BIA: Bio impedance assay
BMI: Body mass index
BMI-Z: Body mass index z score
BP: Blood Pressure
BP-Z: Blood pressure z score
BW: Birth weight
CVD: Cardio vascular disease
DBP: Diastolic blood pressure
DM: Diabetes mellitus
FBS: Fasting blood sugar
FFM: Fat free mass
FFMI: Fat free mass index
FM: Fat mass
FMI: Fat mass index
%FM: Percentage fat mass
HDL-c: High density lipoprotein cholesterol
HOMA-IR: Homeostatic Model Assessment of Insulin Resistance
IOTF: International Obesity Task Force
LDL-c: Low density lipoprotein cholesterol
NCD: Non communicable disease
PPS: Probability-proportionate-to-size
RBS: Random blood sugar
SBP: Systolic blood pressure
SGA: Small for gestational age
TBW: Total body water
TG: Triglyceride
WC: Waist circumference
