# Supplementary material for: The effect of foetal and early childhood growth on metabolic derangements of Sri Lankan children
Source: BMC Pediatr. 2023 Jan 10;23:14. doi: 10.1186/s12887-022-03762-9 (PMC9830816; doi:10.1186/s12887-022-03762-9)
Supplement: Supplementary file 1 — Additional file 1. [file 12887_2022_3762_MOESM1_ESM.docx]

|  | | BW | Z BW | Height | Z Height | Weight | Z Weight^b^ | BMI | Z BMI | FM | FFM | FMI | FFMI | FM% | Waist |
| --- | --- | --- | --- | --- | --- | --- | --- | --- | --- | --- | --- | --- | --- | --- | --- |
| N | | 833 | 833 | 833 | 833 | 833 | 420 | 833 | 833 | 833 | 833 | 833 | 833 | 833 | 833 |
| Mean | | 3.001 | -0.742 | 135.840 | -0.487 | 30.811 | -0.439 | 16.109 | -0.866 | 7.023 | 23.787 | 3.586 | 12.521 | 20.562 | 58.776 |
| Std. Error of Mean | | 0.018 | 0.043 | 0.528 | 0.038 | 0.414 | 0.079 | 0.122 | 0.059 | 0.208 | 0.270 | 0.091 | 0.058 | 0.349 | 0.352 |
| Median | | 3.000 | -0.710 | 134.400 | -0.495 | 28.100 | -0.750 | 15.030 | -1.130 | 4.900 | 22.300 | 2.620 | 12.300 | 17.685 | 56.000 |
| Mode | | 3.000 | -0.520 | 133.000 | -.7900^a^ | 16.700 | -1.3500^a^ | 13.6400^a^ | -1.1300^a^ | 2.900 | 17.7000^a^ | 1.8400^a^ | 11.800 | 14.290 | 48.000 |
| Std. Deviation | | 0.526 | 1.229 | 15.240 | 1.094 | 11.951 | 1.629 | 3.520 | 1.697 | 5.975 | 7.763 | 2.614 | 1.676 | 10.042 | 10.130 |
| Variance | | 0.277 | 1.511 | 232.259 | 1.196 | 142.825 | 2.654 | 12.389 | 2.880 | 35.699 | 60.265 | 6.833 | 2.808 | 100.840 | 102.610 |
| Minimum | | 1.000 | -6.750 | 101.000 | -5.960 | 12.800 | -4.040 | 11.180 | -6.270 | 0.400 | 10.700 | 0.350 | 6.470 | 0.000 | 41.500 |
| Maximum | | 4.800 | 2.940 | 175.300 | 2.380 | 79.800 | 4.960 | 32.890 | 3.590 | 40.300 | 56.900 | 17.060 | 25.470 | 65.300 | 99.000 |
| Percentiles | 25^th^ | 2.700 | -1.405 | 124.700 | -1.185 | 21.350 | -1.630 | 13.640 | -2.135 | 3.100 | 17.850 | 1.855 | 11.495 | 13.433 | 51.000 |
|  | 50^th^ | 3.000 | -0.710 | 134.400 | -0.495 | 28.100 | -0.750 | 15.030 | -1.130 | 4.900 | 22.300 | 2.620 | 12.300 | 17.685 | 56.000 |
|  | 75^th^ | 3.300 | 0.040 | 148.100 | 0.210 | 37.950 | 0.740 | 17.675 | 0.305 | 8.550 | 28.600 | 4.355 | 13.250 | 26.113 | 64.000 |
| ^a^ Multiple modes exist. The smallest value is shown  ^b^ weight for age Z scores were available in WHO 2007 standards for <10 years of age  BMI-Z -BMI z score; BW -birth weight; BW-Z -birth weight Z score; FFM -Fat free mass; FM -Fat mass; FFMI -fat free mass index; FMI -Fat mass index;  Height-Z -Height Z score; %FM -Percentage fat mass; Waist -waist circumference; Weight-Z -Weight z score. | | | | | | | | | | | | | | | |

**Supplementary Table 1a - Descriptive statistics of the anthropometric and body composition parameters of all children of the study sample**

**Supplementary Table 1b - Descriptive statistics of the anthropometric and body composition parameters of 5 -<10-year-old children**

|  | | BW | Z BW | Height | Z Height | Weight | Z Weight | BMI | Z BMI | FM | FFM | FMI | FFMI | FM% | Waist |  |
| --- | --- | --- | --- | --- | --- | --- | --- | --- | --- | --- | --- | --- | --- | --- | --- | --- |
| N | | 420 | 420 | 420 | 420 | 420 | 420 | 420 | 420 | 420 | 420 | 420 | 420 | 420 | 420 |  |
| Mean | | 3.026 | -0.680 | 124.549 | -0.294 | 23.939 | -0.605 | 15.127 | -0.883 | 5.206 | 18.728 | 3.189 | 11.937 | 19.553 | 54.628 |  |
| Std. Error of Mean | | 0.026 | 0.061 | 0.455 | 0.051 | 0.360 | 0.076 | 0.143 | 0.082 | 0.210 | 0.200 | 0.112 | 0.064 | 0.474 | 0.400 |  |
| Median | | 3.000 | -0.595 | 125.000 | -0.310 | 22.100 | -0.860 | 14.165 | -1.180 | 3.500 | 18.200 | 2.320 | 11.760 | 16.495 | 52.000 |  |
| Mode | | 3.000 | -0.730 | 133.000 | -0.790 | 16.7000^a^ | -1.3500^a^ | 13.640 | -2.000^a^ | 2.5000^a^ | 17.700 | 1.6100^a^ | 11.800 | 13.7400^a^ | 48.000 |  |
| Std. Deviation | | 0.531 | 1.252 | 9.332 | 1.044 | 7.384 | 1.502 | 2.923 | 1.674 | 4.285 | 4.086 | 2.279 | 1.316 | 9.685 | 8.170 |  |
| Variance | | 0.282 | 1.567 | 87.092 | 1.090 | 54.530 | 2.255 | 8.543 | 2.803 | 18.362 | 16.695 | 5.195 | 1.731 | 93.805 | 66.747 |  |
| Minimum | | 1.000 | -6.750 | 101.000 | -4.390 | 12.800 | -4.040 | 11.370 | -4.690 | 0.400 | 10.700 | 0.350 | 7.340 | 0.000 | 41.500 |  |
| Maximum | | 4.800 | 2.940 | 152.000 | 2.380 | 50.700 | 3.530 | 30.390 | 3.570 | 24.000 | 35.400 | 12.440 | 20.730 | 52.790 | 87.000 |  |
| Percentiles | 25^th^ | 2.700 | -1.300 | 117.175 | -0.965 | 18.700 | -1.720 | 13.138 | -2.080 | 2.500 | 15.550 | 1.710 | 11.200 | 12.900 | 49.000 |  |
|  | 50^th^ | 3.000 | -0.595 | 125.000 | -0.310 | 22.100 | -0.860 | 14.165 | -1.180 | 3.500 | 18.200 | 2.320 | 11.760 | 16.495 | 52.000 |  |
|  | 75^th^ | 3.400 | 0.150 | 131.175 | 0.405 | 27.325 | 0.405 | 16.150 | 0.260 | 6.400 | 20.900 | 3.960 | 12.540 | 24.285 | 58.000 |  |
| ^a^ Multiple modes exist. The smallest value is shown  BMI-Z -BMI z score; BW -birth weight; BW-Z -birth weight Z score; FFM -Fat free mass; FM -Fat mass; FFMI -fat free mass index; FMI -Fat mass index;  Height-Z -Height Z score; %FM -Percentage fat mass; Waist -waist circumference; Weight-Z -Weight z score. | | | | | | | | | | | | | | | | |

**Supplementary Table 1c - Descriptive statistics of the anthropometric and body composition parameters of 10 - 15-year-old children**

|  | | BW | BW-Z | Height | Z Height | Weight | BMI | Z BMI | FM | FFM | FMI | FFMI | FM% | Waist |
| --- | --- | --- | --- | --- | --- | --- | --- | --- | --- | --- | --- | --- | --- | --- |
| N | | 413 | 413 | 413 | 413 | 413 | 413 | 413 | 413 | 413 | 413 | 413 | 413 | 413 |
| Mean | | 2.976 | -0.805 | 147.323 | -0.682 | 37.799 | 17.109 | -0.849 | 8.862 | 28.908 | 3.988 | 13.112 | 21.587 | 62.994 |
| Std. Error of Mean | | 0.026 | 0.059 | 0.535 | 0.055 | 0.574 | 0.186 | 0.085 | 0.336 | 0.356 | 0.141 | 0.088 | 0.508 | 0.503 |
| Median | | 3.000 | -0.750 | 148.200 | -0.620 | 36.500 | 16.030 | -1.080 | 6.500 | 28.250 | 3.000 | 12.965 | 18.845 | 61.000 |
| Mode | | 2.800 | -0.520 | 156.000 | -1.760^a^ | 27.800^a^ | 14.650 | -1.600^a^ | 6.400 | 32.300 | 1.600^a^ | 11.730^a^ | 19.380^a^ | 60.000 |
| Std. Deviation | | 0.521 | 1.204 | 10.877 | 1.109 | 11.657 | 3.787 | 1.722 | 6.826 | 7.228 | 2.861 | 1.791 | 10.302 | 10.196 |
| Variance | | 0.271 | 1.450 | 118.305 | 1.231 | 135.876 | 14.345 | 2.966 | 46.594 | 52.246 | 8.186 | 3.209 | 106.134 | 103.955 |
| Minimum | | 1.000 | -6.680 | 110.500 | -5.960 | 16.700 | 11.180 | -6.270 | 0.700 | 11.400 | 0.390 | 6.470 | 2.870 | 47.000 |
| Maximum | | 4.600 | 2.580 | 175.300 | 2.070 | 79.800 | 32.890 | 3.590 | 40.300 | 56.900 | 17.060 | 25.470 | 65.300 | 99.000 |
| Percentiles | 25^th^ | 2.700 | -1.420 | 139.500 | -1.305 | 28.800 | 14.495 | -2.155 | 4.300 | 23.700 | 2.083 | 11.993 | 13.925 | 55.000 |
|  | 50^th^ | 3.000 | -0.750 | 148.200 | -0.620 | 36.500 | 16.030 | -1.080 | 6.500 | 28.250 | 3.000 | 12.965 | 18.845 | 61.000 |
|  | 75^th^ | 3.300 | -0.070 | 155.300 | 0.045 | 43.900 | 18.930 | 0.330 | 11.350 | 33.100 | 4.838 | 14.020 | 27.415 | 68.000 |
| ^a^ Multiple modes exist. The smallest value is shown  BMI-Z -BMI z score; BW -birth weight; BW-Z -birth weight Z score; FFM -Fat free mass; FM -Fat mass; FFMI -fat free mass index; FMI -Fat mass index;  Height-Z -Height Z score; %FM -Percentage fat mass; Waist -waist circumference; Weight-Z -Weight z score. | | | | | | | | | | | | | | |
